# Clinical significance and diagnostic value of serum CEA, CA19-9 and CA72-4 in patients with gastric cancer

**DOI:** 10.18632/oncotarget.10391

**Published:** 2016-07-02

**Authors:** Yao Liang, Wei Wang, Cheng Fang, Seeruttun Sharvesh Raj, Wan-Ming Hu, Qi-Wen Li, Zhi-Wei Zhou

**Affiliations:** ^1^ Department of Gastric and Pancreatic Surgery, Sun Yat-sen University Cancer Center, State Key Laboratory of Oncology in South China, Collaborative Innovation Center for Cancer Medicine, Guangzhou, Guangdong, China; ^2^ Department of Pathological Oncology, Sun Yat-sen University Cancer Center, State Key Laboratory of Oncology in South China, Collaborative Innovation Center for Cancer Medicine, Guangzhou, Guangdong, China; ^3^ Department of Radiation Oncology, Sun Yat-sen University Cancer Center, State Key Laboratory of Oncology in South China, Collaborative Innovation Center for Cancer Medicine, Guangzhou, Guangdong, China

**Keywords:** CEA, CA19-9, CA72-4, diagnosis, gastric cancer

## Abstract

**Aims:**

To evaluate the clinical significance of multiple serum tumor markers (TMs) in the diagnosis of gastric cancer (GC) and establish an accurate discriminant equation to identify the presence of GC.

**Results:**

The serum levels of CEA, CA19-9 and CA72-4 were higher in the GC group than in the control group (*P* < 0.005). The sensitivity of CEA, CA19-9 and CA72-4 in the diagnosis of GC was 20.1–27.6% individually and increased to 48.2% when they were considered in combination. By using the optimal cut-off value, the sensitivity of CEA, CA19-9 and CA72-4 for the diagnosis of GC was improved but remained unsatisfactory. In addition, we developed the equation Y = −2.185 − 0.015 X1 + 0.180 X2 + 1.226 X3 + 1.505 X4 + 2.749 X5 (X1 = Age, X2 = Sex, X3 =CEA, X4 = CA19-9 and X5 = CA72-4) to predict the presence of GC. This has better accuracy and diagnostic efficiency compared to the combination of TMs.

**Methods:**

Serum carcinoembryonic antigen (CEA), cancer antigen 19-9 (CA19-9)and cancer antigen 72-4 (CA72-4) levels were measured in a total of 2288 patients with GC and 1869 healthy volunteers or patients with benign gastric diseases. We established a diagnostic equation using a portion of the data (training set), and validate its accuracy using the other portion of the data (testing set).

**Conclusions:**

The diagnostic equation increases the accuracy rate for the diagnosis of GC and will be helpful in the clinic.

## INTRODUCTION

Despite decreasing incidence and mortality worldwide, [1, 2] gastric cancer (GC) remains the third most prevalent cancer and third leading cause of cancer-related death in mainland China. [3] The estimated number of new cases and deaths in China is much higher than in any other country and comprises nearly one-half of the global total. [4, 5] The high mortality rate is mainly attributed to late detection. In particular, the proportion of early GC detection was only 9%, even in many high-volume hospitals in China. [6] Therefore, we need invent an easy and quick method for screening for the disease.

Endoscopic biopsy and histopathological evaluation are considered the gold standard for diagnosing GC, but the stress caused by this method and its high expense make it difficult to use it as a routine method for screening on a population basis, particularly for asymptomatic individuals. [7, 8] The detection of serum indicators is simple and easy and has become a common clinical method for screening tumors. Tumor markers (TMs), such as alpha fetoprotein (AFP), carcinoembryonic antigen (CEA), cancer antigen 19-9 (CA19-9) and cancer antigen 72-4 (CA72-4), have been widely used for the diagnosis of different types of cancers, including liver, colorectal cancer and pancreatic cancers. [9–11] However, according to the current knowledge, most previous studies on TMs focused on their use as prognostic indicators, predicting the efficacy of both first-line chemotherapy and postsurgical surveillance in GC. [12–14] There are few studies on predictive screening or early detection, particularly for CA72-4.

Therefore, the objective of the present study was to investigate the diagnostic value of serum TMs for GC. Moreover, we explored the relationship between the TM levels and the clinicopathological features of GC and established an accurate discriminant equation to predict the occurrence of GC.

## RESULTS

### Serum TMs in the training set

**Figure 1:**
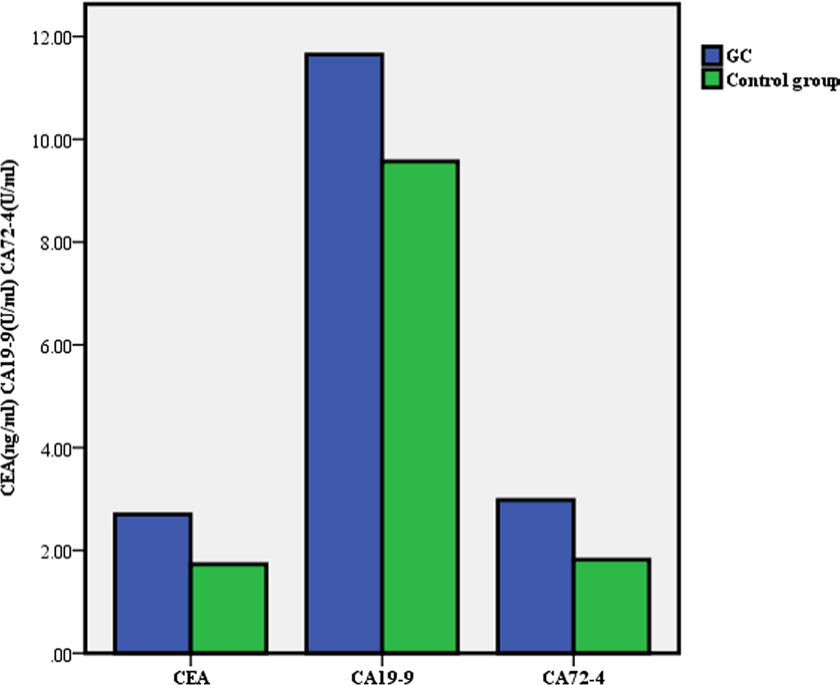
Mean levels of carcinoembryonic antigen, cancer antigen 19-9 and cancer antigen 72-4 in the gastric cancer (GC) group and control group respectively

Statistical data, including age and gender, and the test results of the TMs in the two groups are shown in Table 1. The median serum concentrations and positive rates of CEA, CA19-9 and CA72-4 were significantly higher in the GC group than in the control group (Figure 1).

**Table 1:** Serum levels and positive rates of carcinoembryonic antigen, cancer antigen 19-9 and cancer antigen 72-4 in the training set

	GC	Control group	Total	*P*
Cases	1945	1589	3534	
Age(y)	55.83 ± 11.988	55.99 ± 10.218	55.91 ± 11.225	0.672
Gender(M/F)	1304/641	1073/516	2377/1157	0.761
CEA(ng/ml)	2.7(2.59)	1.73(1.54)	2.21(2.02)	< 0.001
CA19-9(U/ml)	11.65(16.52)	9.57(8.08)	10.38(11.31)	< 0.001
CA72-4(U/ml)	2.98(3.79)	1.82(2.28)	2.54(2.96)	< 0.001
CEA positive cases(%)	390(20.1%)	84(5.3%)	473(13.4%)	< 0.001
CA19-9 positive cases(%)	416(21.4%)	60(3.8%)	475(13.4%)	< 0.001
CA72-4 positive cases(%)	537(27.6%)	235(14.8%)	771(21.8%)	< 0.001
Combination positive cases(%)	938(48.2%)	346(21.8%)	1284(36.3%)	< 0.001

GC = gastric cancer group, CEA = carcinoembryonic antigen, CA19-9 = cancer antigen 19-9, CA72-4 = cancer antigen 72-4.

### Positive rates of serum TMs in GCs with different clinicopathological features

**Figure 2:**
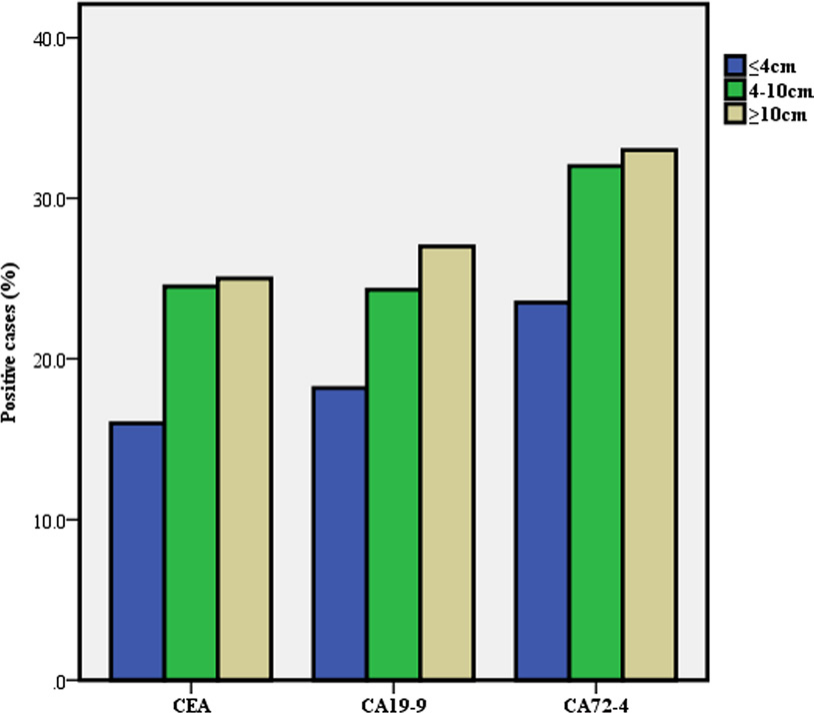
Positive rate of serum tumor markers in gastric cancer according to tumor size

**Figure 3:**
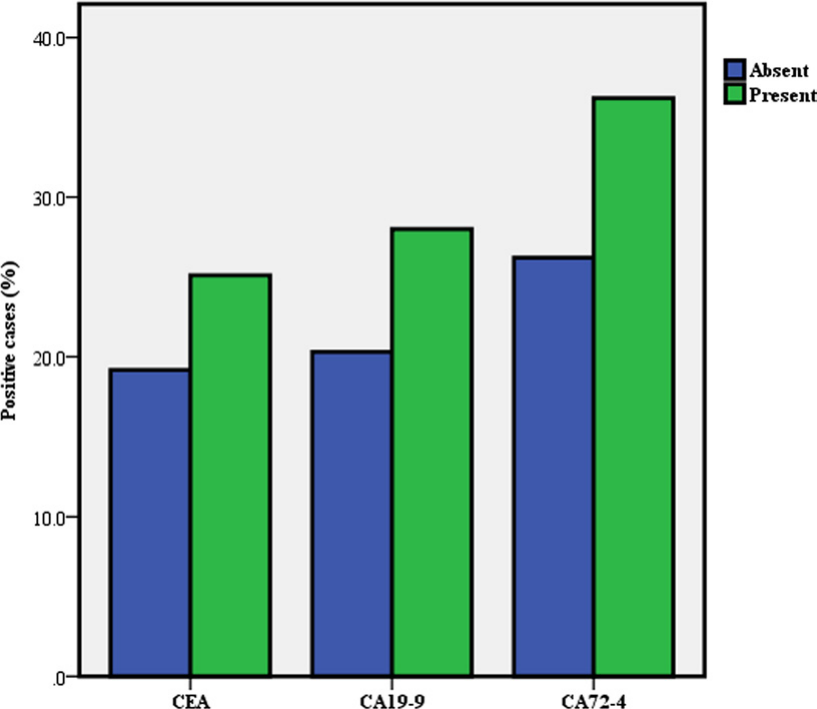
Positive rate of serum tumor markers in gastric cancer according to vascular embolism

**Figure 4:**
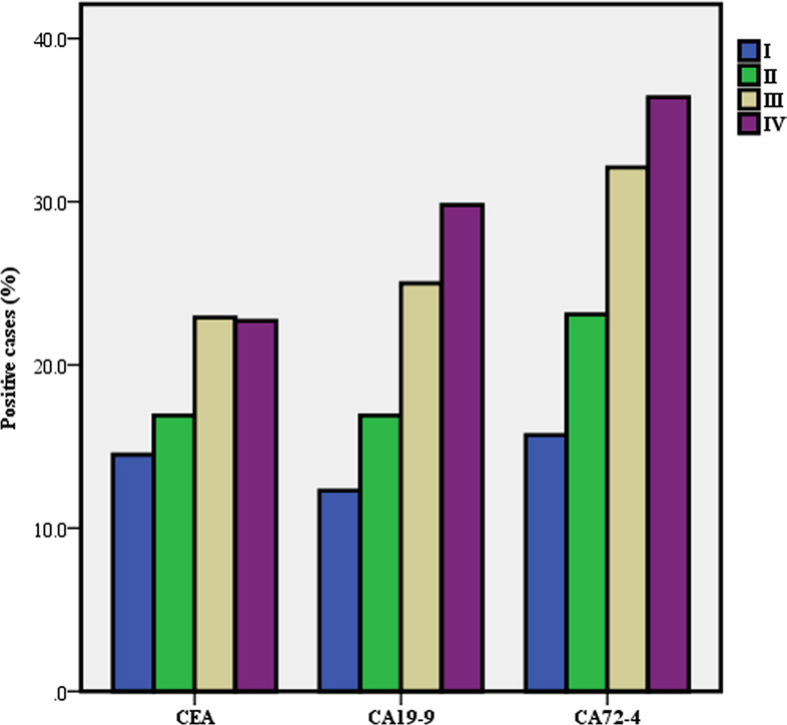
Positive rate of serum tumor markers in gastric cancer according to stage

According to the baseline information for GC, we subdivided the GC group and calculated the positive rates of the three TMs in each group (Table 2). We found that there were significant differences in the positive rates of TMs among GCs that correlated to the differences in tumor size, vascular embolism, wall invasion, nodal metastases and stage (Figures 2, 3, 4).

**Table 2:** Relationship between serum tumor markers and clinicopathological characteristics of the gastric cancer group n(%)

	Cases(%)	Positive cases (%)					
		CEA	*P*	CA19-9	*P*	CA72-4	*P*
Sex							
male	1304(67)	293(22.5)	0.000	268(20.6)	0.200	368(28.2)	0.389
female	641(33)	97(15.1)		148(23.1)		169(26.4)	
Age(yr)							
< 60	1155(59.4)	186(16.1)	0.000	239 (20.7)	0.366	308(26.6)	0.261
≥ 60	790(40.6)	204(25.8)		177(22.4)		229(29.0)	
Tumor size(cm)							
≤ 4	1026(52.8)	164(16.0)	0.001	187(18.2)	0.001	241(23.5)	0.000
4 – 10	719(37)	176(24.5)^a^		175(24.3)^a^		230(32.0)^a^	
≥ 10	200(10.3)	50(25.0)^a^		54(27.0)^a^		66(33.0)^a^	
Location							
cardia	472(24.3)	121(25.6)	0.005	114(24.2)	0.112	137(29.0)	0.167
gastric body	391(20.1)	75(19.2)^b^		82(21)		103(26.3)	
gastric antrum	970(49.9)	176 (18.1)^b^		190(19.6)		257(26.5)	
multi-site	112(5.8)	18(16.1)^b^		30(26.8)		40(35.7)	
Differentiation							
high /moderate	376(19.3)	108(28.7)	0.000	81(21.5)	0.211	102(27.1)	0.034
poor	1159(59.6)	216(18.6)^c^		232(20)		300(25.9)	
signet ring	373(19.2)	58(15.5)^c^		96(25.7)		127(34.0)^c^^d^	
other	37(1.9)	8(21.6)		7(18.9)		8(21.6)	
Vascular embolism							
present	271(13.9)	68(25.1)	0.025	76(28.0)	0.004	98(36.20)	0.001
absent	1674(86.1)	322(19.2)		340(20.3)		439(26.2)	
Wall invasion							
T1	273(14)	42(15.4)	0.001	32(11.7)	0.000	44(16.1)	0.000
T2	209(10.7)	31(14.8)		27(12.9)		43(20.6)^e^	
T3	334(17.2)	55(16.5)		63(18.9)^e^		89(26.6)^e^	
T4a	920(47.3)	208(22.6)^e^		235(25.5)^e^		290(31.5)^e^	
T4b	209(10.7)	54(25.8)^e^		59(28.2)^e^		71(34.0)^e^	
Nodal metastases							
N0	601(30.9)	94(15.6)	0.000	88(14.6)	0.000	114(19)	0.000
N1	322(16.6)	50(15.5)		80(24.8)^f^		87(27)^f^	
N2	350(18)	91(26)^f^^g^		78(22.3)^f^		107(30.6)^f^	
N3	672(34.6)	155(23.1)^f^^g^		170(25.3)^f^		229(34.1)^f^^g^	
Metastases							
M0	1719(88.4)	338(19.7)	0.238	349(20.3)	0.001	455(26.5)	0.002
M1	226(11.6)	52(23.0)		67 (29.6)		82(36.3)	
Stage							
I	351(18.0)	51(14.5)	0.003	43(12.3)	0.000	55(15.7)	0.000
II	438(22.5)	74(16.9)		74(16.9)		101(23.1)^h^	
III	931(47.8)	214(22.9)^h^^i^		232(25.0)^h^^i^		299(32.1)^h^^i^	
IV	225(11.6)	51(22.7)^h^		67(29.8)^h^^i^		82(36.4)^h^^i^	

a Statistically significant compared to ≤ 4 subgroup

b Statistically significant compared to cardia subgroup

c Statistically significant compared to high/moderate subgroup

d Statistically significant compared to poor subgroup

e Statistically significant compared to T1 subgroup

f Statistically significant compared to N0 subgroup

g Statistically significant compared to N1 subgroup

h Statistically significant compared to I subgroup

i Statistically significant compared to II subgroup

Stratified analysis showed that the positive rate of CEA in cardia-located GC was significantly higher than that of GC in other locations. Conversely, no difference was found among the location groups for CA19-9 and CA72-4. The differences in differentiation subgroups were significant for CEA and CA72-4 but not for CA19-9. Additionally, the positive rates of the three TMs increased with tumor stage, and statistically significant differences were found between stages III, IV and I for CEA and between stages III and IV and stages I and II for CA19-9 and CA72-4, respectively.

### Use of normal or optimum cut-off values of serum TMs for the diagnosis of GC

We used the normal reference value of TMs as the cut-off value to determine the negative and positive GCs. The results showed that the use of a single serum TM had good specificity (85.1%–96.2%) but poor sensitivity (20.1%–27. 6%) for the diagnosis of GC. The sensitivity was improved to 48.2% with the combined use of serum TMs, but it remained unsatisfactory (Table 3).

**Table 3:** Use of normal cut-off values of serum tumor markers for gastric cancer diagnosis

	CEA (ng/ml)	CA19-9 (U/ml)	CA72-4 (U/ml)	CEA+CA199	CEA+CA724	CA724+CA199	Combination
Normal boundary value	≤ 5	≤ 27	≤ 5.3				
AUC(95%CI)	0.712	0.585	0.722	0.634	0.609	0.609	0.632
	0.695–0.729	0.566–0.603	0.704–0.740	0.616–0.652	0.591–0.628	0.591–0.628	0.614–0.650
Sensitivity(%)	20.10	21.40	27.60	35.10	40.10	40.10	48.20
Specificity (%)	94.70	96.20	85.10	91.80	81.70	81.70	78.20
PPV/NPV(%)	82.3/49.2	87.4/50.0	69.5/49.0	83.9/53.6	72.9/52.7	72.9/52.7	73.1/55.2
PLR/NLR	3.79/0.85	5.63/0.82	1.85/0.85	4.28/0.71	2.19/0.73	2.19/0.73	2.21/0.66
Acurancy (%)	53.62	55.04	53.45	60.58	58.80	58.80	53.45
YI	0.15	0.18	0.13	0.27	0.22	0.22	0.26

AUC = area under curve, CI = confidence interval, PPV = positive predictive value, NPV = negative predictive value, PLR = positive likelihood ratio, NLR = negative likelihood ratio, YI = Youden's Index.

**Figure 5:**
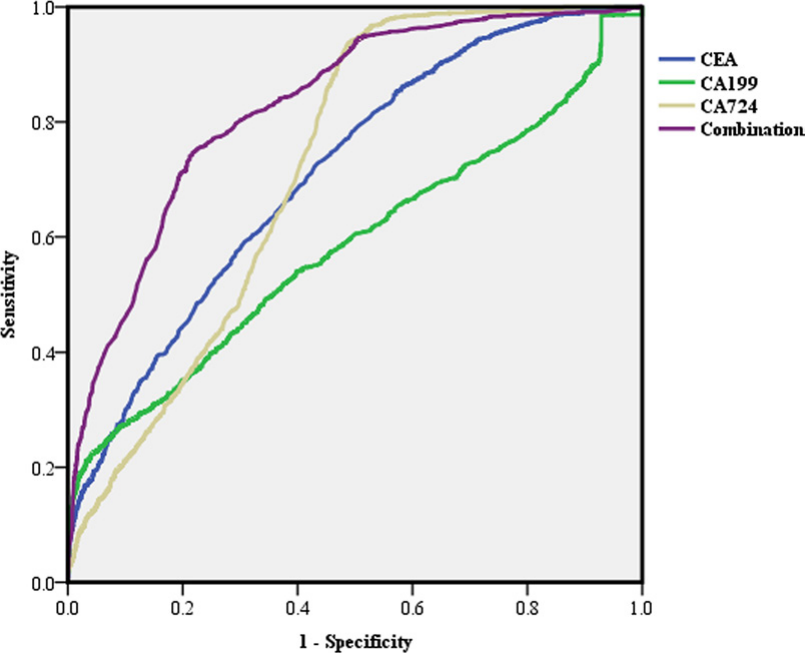
Receiver operating characteristic (ROC) curve of single and combined tumor markers with the optimum cut-off values in predicting gastric cancer (GC)

Because the low sensitivities with the use of normal cut-off values restrict their clinical application, we analyzed the AUC of the ROC curve to obtain the optimum diagnostic cut-off values of the serum TMs and then calculated their diagnostic capacities for GC (Table 4). The analysis showed that CA72-4 was the preferable single test, with a sensitivity value (93.83%) that was higher than that of CEA (72.20%) and much higher than that of CA19-9 (22.30%). In addition, the diagnostic specificity for the CA19-9 levels (95.9%) was higher than that of the other TMs. The use of optimum boundary values significantly improved the diagnostic efficiency of each single marker for GC, which remained lower than the combination (*P* < 0.05), according to the ROC curve (Figure 5).

**Table 4:** Use of optimal cut-off values of serum tumor markers for gastric cancer diagnosis

	CEA (ng/ml)	CA19-9 (U/ml)	CA72-4 (U/ml)	CEA+CA199	CEA+CA724	CA724+CA199	Combination
Optimal boundary value	≥ 1.93	≥ 26.18	≥ 1.84				
AUC(95%CI)	0.712	0.585	0.722	0.657	0.725	0.725	0.735
	0.695–0.729	0.566–0.603	0.704–0.740	0.639–0.675	0.707–0.742	0.707–0.742	0.718–0.752
Sensitivity(%)	72.20	22.30	93.83	75.60	93.80	93.80	73.60
Specificity (%)	57.00	95.90	51.10	55.80	51.10	51.10	76.00
PPV/NPV(%)	67.3/62.7	86.9/50.2	70.1/87.1	67.7/65.1	70.1/87.1	70.1/87.1	83.7/63.2
PLR/NLR	1.68/0.49	5.44/0.81	1.92/0.12	1.71/0.44	1.92/0.12	1.92/0.12	3.07/0.35
Acurancy (%)	65.39	55.38	74.59	66.69	74.59	74.59	74.48
YI	0.29	0.18	0.45	0.31	0.45	0.45	0.50

AUC = area under curve, CI = confidence interval, PPV = positive predictive value, NPV = negative predictive value, PLR = positive likelihood ratio, NLR = negative likelihood ratio, YI = Youden's Index.

**Figure:**
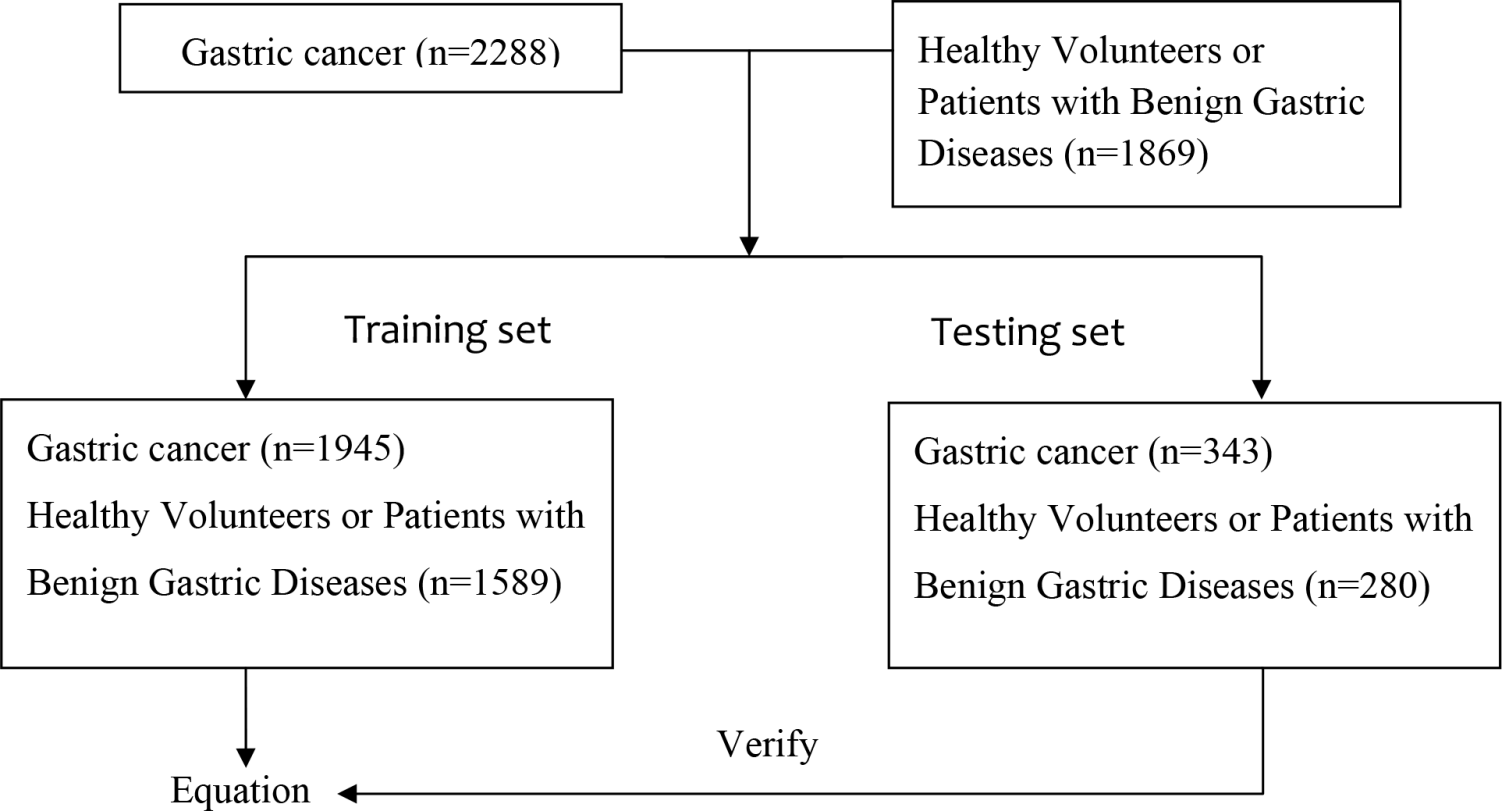


### Establishment and validation of the criterion equation for GC diagnosis

To establish an accurate method for evaluating the possibility of GC by using serum TMs, we obtained a classification discriminant equation using the multivariate logistic regression analysis to ascertain whether patients have GC as follows: Y = −2.185–0.015×1+0.180×2+ 1.226×3+1.505×4+2.749×5 (where X1 = Age, X2 = Sex, X3 = CEA, X4 = CA19-9 and X5 = CA72-4), for which the critical value is 0.50, thus, if the Y of a case is larger than 0.50, it belongs to the GC group. The values for all parameters are shown in Table 5.

**Table 5:** The values for all parameters of the equation

Parameter		Values
X1 = Age	Calculate by continuous variable	
X2 = Sex	Female	0
	Male	1
X3 = CEA	< 1.93 ng/ml	0
	≥ 1.93 ng/ml	1
X4 = CA19-9	< 26.18 U/ml	0
	≥ 26.18 U/ml	1
X5 = CA72-4	< 1.84 U/ml	0
	≥ 1.84 U/ml	1

Then, we verified the discriminant equation model using the data of the testing set (343 GC patients and 280 healthy volunteers or patients with benign gastric diseases). There were 49 healthy cases mistaken for GC and 22 GC patients whose diagnoses were missed by the equation results(Table 6). The sensitivity, specificity, PPV and NPV were 93.59%, 82.5%, 86.76% and 91.3%, respectively. Youden's index was 0.76, and the accuracy was 88.60, which was statistically higher than the combination of TMs described above (χ^2^ = 452.5, *P* < 0. 001).

**Table 6:** The discriminant value of testing set

	Gold standard		
Results of criterion equation	+	–	Total
Positive	321	49	370
Negative	22	231	253
Total	343	280	623

## DISCUSSION

An estimated 951,600 new stomach cancer cases and 723,100 deaths occurred in 2012. [15] Early spread to metastatic sites is considered the main reason for the high death rate, and early diagnosis may improve the long-term survival rates and reduce the mortality from this disease. Therefore, it is necessary to develop a convenient diagnostic method for routine screening, which would increase the early diagnosis of GC. TMs have been widely used in the domain of cancer. However, each TM has its limitation in terms of diagnostic value, particularly for early diagnosis. [16] It is therefore necessary to identify new combination models of TMs.

In this study, we found that the serum levels and positive rates of CEA, CA19-9 and CA72-4 in the GC group were higher than those in the control group, which is consistent with a previous report, [17] and our data suggest that these markers have diagnostic value for GC.

Furthermore, the relationship between serum TMs and the clinicopathological features of GC was investigated. We found that the positive rates of CEA, CA19-9 and CA72-4 increased with tumor stage. This trend indicates that both the progression and burden of the tumor affect the TMs. These findings are consistent with earlier studies. [13, 18]

Then, we evaluated the diagnostic efficiency of TMs for GC. Regardless of whether we used the normal reference values or optimum boundary values of our markers as a cut-off value to assess the clinical specimens, we found that the sensitivity of all TMs was very low, whereas the combination of TMs could improve the sensitivity, though it remained unsatisfactory. These results are consistent with previous studies performed within the Chinese population. [19]

To obtain a better pattern to improve the accuracy of GC detection, we developed the discriminant equation according to a multivariate logistic regression analysis. The results from the testing set demonstrate that this equation could distinguish GC patients from healthy controls, and it has a better diagnostic power than the CEA+CA19-9+CA72-4 pattern. Using this equation to diagnose GC can achieve a higher sensitivity, specificity, and accuracy and can be practically applied in clinical practice.

Currently, the NCCN Clinical Practice Guideline in Gastric Cancer 2015 and ESMO Clinical Recommendations for Gastric Cancer 2014 have not discussed any tumor biomarkers or combined biomarkers for GC for early screening and diagnosis. [20, 21] We hope to provide a useful reference for the application of TMs for GC in the future.

In conclusion, TMs such as CEA, CA19-9, and CA72-4 show a correlation with the diagnosis of GC. The discriminant equation may be a useful tool for the prediction of GC.

## MATERIALS AND METHODS

### Study subjects

A total of 4157 subjects (2288 GC patients and 1869 healthy volunteers or patients with benign gastric diseases) visiting Sun Yat-sen University Cancer Center from January 2000 to May 2015 were enrolled in the study. From these patients, we simultaneously chose a group of subjects (343 and 280, respectively) as a testing set to verify the discriminant equation model. The remainder of the subjects comprised the training set including the GC group and control group (*n* = 1945 and *n* = 1589, respectively). In the GC group, there were 1304 males and 641 females, who ranged in age from 16 to 86 years, with an average age of 55.58 years. Among them, 1159 patients had poorly differentiated adenocarcinoma, 376 patients had moderately or highly differentiated adenocarcinoma, 970 patients had gastric antrum carcinoma, 391 patients had gastric body cancer, 472 patients had gastric cardia cancer, and 112 patients had multiple site cancer (more than two sites). In the control group, there were 1073 males and 516 females, with an age range of 5 to 83 years, and an average age of 45.99 years.

Patients with GC were diagnosed by endoscopy and confirmed by biopsy, and did not receive any preoperative treatment. The staging of cancer was based on a routine histopathological analysis and clinical assessment, according to the 7th AJCC (American Joint Committee on Cancer) Gastric Cancer TNM Staging System. [22]

### Ethical considerations

The Medical Ethics Committee of Sun Yat-sen University Cancer Center approved this study. All subjects provided written informed consent to offer related information in the hospital.

### Serum TM detection

Blood samples were collected prior to any therapy in GC patients and as part of a routine examination in control subjects. Serum CEA, CA19-9, CA72-4 levels were measured using Cobas 601 and reagent kits (Roche Diagnostics, Mannheim, Germany) at the clinical laboratory of the Sun Yat-sen University Cancer Center. The cut-off values of 5 ng/ml, 27 U/ml and 5.3 U/ml for CEA, CA19-9 and CA72-4, respectively, were used.

### Statistical analysis

The χ^2^ test was used to analyze the statistical significance of categorical variables, and the *T*-test was used for continuous variables. The area under the curve (AUC) of the receiver operating characteristic (ROC) curve was used to evaluate the diagnostic value of serum TMs. The diagnosis discriminant equation for GC was established based on the multivariate logistic regression analysis. The differences were considered statistically significant when *P* < 0.05. All analyses were performed using SPSS version 20.0 (SPSS Inc., Chicago, IL, USA).
